# Global Inhibition of Reactive Oxygen Species (ROS) Inhibits Paclitaxel-Induced Painful Peripheral Neuropathy

**DOI:** 10.1371/journal.pone.0025212

**Published:** 2011-09-26

**Authors:** Mehmet Fidanboylu, Lisa A. Griffiths, Sarah J. L. Flatters

**Affiliations:** Wolfson Centre for Age-Related Diseases, Centre for Integrative Biomedicine, King's College London, London, United Kingdom; University of Cincinnatti, United States of America

## Abstract

Paclitaxel (Taxol®) is a widely used chemotherapeutic agent that has a major dose limiting side-effect of painful peripheral neuropathy. Currently there is no effective therapy for the prevention or treatment of chemotherapy-induced painful peripheral neuropathies. Evidence for mitochondrial dysfunction during paclitaxel-induced pain was previously indicated with the presence of swollen and vacuolated neuronal mitochondria. As mitochondria are a major source of reactive oxygen species (ROS), the aim of this study was to examine whether pharmacological inhibition of ROS could reverse established paclitaxel-induced pain or prevent the development of paclitaxel-induced pain. Using a rat model of paclitaxel-induced pain (intraperitoneal 2 mg/kg paclitaxel on days 0, 2, 4 & 6), the effects of a non-specific ROS scavenger, N-tert-Butyl-α-phenylnitrone (PBN) and a superoxide selective scavenger, 4-hydroxy-2,2,6,6-tetramethylpiperidine-1-oxyl (TEMPOL) were compared. Systemic 100 mg/kg PBN administration markedly inhibited established paclitaxel-induced mechanical hypersensitivity to von Frey 8 g and 15 g stimulation and cold hypersensitivity to plantar acetone application. Daily systemic administration of 50 mg/kg PBN (days −1 to 13) completely prevented mechanical hypersensitivity to von Frey 4 g and 8 g stimulation and significantly attenuated mechanical hypersensitivity to von Frey 15 g. Systemic 100 mg/kg TEMPOL had no effect on established paclitaxel-induced mechanical or cold hypersensitivity. High dose (250 mg/kg) systemic TEMPOL significantly inhibited mechanical hypersensitivity to von Frey 8 g & 15 g, but to a lesser extent than PBN. Daily systemic administration of 100 mg/kg TEMPOL (day −1 to 12) did not affect the development of paclitaxel-induced mechanical hypersensitivity. These data suggest that ROS play a causal role in the development and maintenance of paclitaxel-induced pain, but such effects cannot be attributed to superoxide radicals alone.

## Introduction

Paclitaxel is a taxane-derived chemotherapeutic used alone, or in combination therapy, for the treatment of ovarian, breast and advanced non-small cell lung cancers, and AIDS-related Kaposi's sarcoma. Paclitaxel binds to β-tubulin of microtubules [1], stabilising microtubules and interfering with spindle-microtubule dynamics, arresting mitosis and inducing apoptosis [2]. Painful peripheral neuropathy is the major dose-limiting side-effect of paclitaxel therapy. Patients describe various sensory symptoms including mechanical allodynia, spontaneous pain, cold allodynia, numbness, tingling, in a ‘stocking and glove’ distribution [3], [4], [5]. Emergence of these symptoms can mean that patients cannot complete optimal chemotherapy schedules [6] thus potentially limiting anti-cancer actions. The incidence and severity of paclitaxel-induced pain symptoms correlates with increasing cumulative doses of paclitaxel [7], [8]. Following the cessation of paclitaxel, pain and sensory abnormalities can persist for months or years [5], [9]. Currently, there is no effective therapy for the prevention or treatment of chemotherapy-induced painful peripheral neuropathy. Several analgesics with established efficacy in other painful neuropathies have failed to show any efficacy in double-blinded, placebo-controlled trials of patients with chemotherapy-induced painful peripheral neuropathy [10], [11], [12], [13].

Reactive oxygen species (ROS) e.g. superoxide radical O_2_^−^, hydroxyl radical OH^.^, are by-products of oxidative phosphorylation and usually decomposed by specialised cellular enzymes e.g.superoxide dismutases, peroxidases. In the 1990s, the role of ROS in neuropathic pain was demonstrated with the inhibition of CCI-evoked heat hyperalgesia by novel antioxidants 4-hydroxy-2,2,6,6-tetramethylpiperidine-1-oxyl (TEMPOL) [14], N-acetyl-cysteine [15] and tirilazad [16] and increased superoxide dismutase levels in the axotomised sciatic nerve [17]. More recently, pharmacological inhibition of ROS was reported to have anti-nociceptive effects in neuropathic and inflammatory pain models. N-tert-Butyl-α-phenylnitrone (PBN), a non-specific ROS scavenger, inhibited mechanical hypersensitivity evoked by spinal nerve ligation (SNL) [18], [19], [20], capsaicin-induced inflammation [21], [22] and visceral inflammation [23]. Other non-specific ROS scavengers, 5,5-dimethylpyrroline-N-oxide and nitrosobenzene also relieved neuropathic pain behaviours [18]. Reagents that mimic superoxide dismutase activity (thus specifically scavenging superoxide), inhibited hypersensitivity to mechanical/heat stimuli evoked by either peripheral nerve injury [14], [20] or inflammation [21], [22], [24], [25]. Furthermore, mitochondrial ROS-producing profiles are increased in the spinal cord following peripheral nerve injury [26] or an inflammatory stimulus [22], [27].

Our interest in the role of ROS in chemotherapy-induced pain developed after finding swollen/vacuolated mitochondria in peripheral sensory nerves of paclitaxel-treated rats, in the absence of axonal degeneration [28]. These atypical changes in neuronal mitochondria correlated with the paclitaxel-induced pain time course i.e. present during the pain syndrome but not at its resolution [28]. Considering that mitochondria are a major source of ROS as a by-product of oxidative phosphorylation, we examined the potential causal role of ROS in chemotherapy-induced painful peripheral neuropathy. Here we compare the effects of systemic administration of two ROS scavengers with differing selectivity, PBN and TEMPOL, on paclitaxel-induced pain. Using a rat model of paclitaxel-induced pain, we assess the ability of PBN, a non-specific ROS scavenger and TEMPOL, a superoxide dismutase mimetic, to a) inhibit established paclitaxel-induced pain and b) counteract the development of paclitaxel-induced pain.

## Methods

All experiments were carried out in strict accordance with the UK Animals (Scientific Procedures) Act 1986 and the ethical guidelines issued by the International Association for the Study of Pain [29]. The protocol was approved by the Ethical Review Panel of King's College London and conducted under the UK Home Office project license 70/6673. Adult male Sprague-Dawley rats (starting weight 180–220 g, Harlan/Charles-River, UK) were housed in groups of 3–4 on sawdust bedding in plastic cages with environmental enrichment materials. Artificial lighting was provided on a fixed 12 h light/12 h dark cycle (7am lights on) with food and water available *ad libitum*. Bedding/cages were changed twice a week and only rats were housed in the same room. Prior to any behavioural testing, rats were habituated to the testing environment for 30 minutes on two or three separate days.

### 2.1 Administration of paclitaxel

Following habituation to the behavioural testing environment and baseline measurements of mechanical sensitivity (see Section 2.2), rats were injected intraperitoneally (i.p.) with 2 mg/kg paclitaxel on four alternate days (days 0, 2, 4 and 6) as previously described [28], [30], [31], [32]. 2 mg/ml paclitaxel was prepared with sterile 0.9% saline for injection (Fresenius Kabi, UK) from the clinical formulation of 6 mg/ml Paclitaxel Concentrate for Solution for Infusion (CP Pharmaceuticals Ltd, UK).

### 2.2 Behavioural assessment of mechanical hypersensitivity

Animals were placed on an elevated platform of small metal rods (spaced 8 mm apart) in individual Perspex boxes (dimensions 15 cm×16 cm×21 cm). Animals were allowed to acclimatise for 5–10 minutes before testing. Mechanical hypersensitivity was assessed using three von Frey filaments with bending forces of 4 g, 8 g and 15 g, in ascending order of force, as previously described [28], [31], [32]. Each application of a von Frey filament to the hind paw was held for five seconds and each hind paw was stimulated five times with each of the three von Frey filaments. The application of each filament was varied within the mid-plantar area to avoid stimulating the footpads or the same spot twice. Withdrawal responses to the von Frey filaments from both hind paws were counted and combined to give an overall percentage response, e.g. if a rat withdrew to 4 out of the10 applications of von Frey 8 g, this was recorded as 40% overall response to von Frey 8 g for that rat. All testing was performed on rats when they were alert, not grooming and with all four paws in contact with the platform. Following habituation, three baseline measurements of mechanical sensitivity were taken prior to paclitaxel administration and averaged.

### 2.3 PBN experiments on mechanical hypersensitivity

N-tert-Butyl-α-phenylnitrone (PBN, Sigma-Aldrich, UK) was dissolved in sterile 0.9% saline for injection (Fresenius Kabi, UK) resulting in a clear, colourless solution. PBN was administered intraperitoneally (i.p.) in either a treatment or prophylactic dosing paradigm, to test if PBN could treat established paclitaxel-induced pain or prevent the development of paclitaxel-induced pain.

#### Treatment dosing paradigm

Following habituation and baseline testing, all rats received paclitaxel as described above and the emergence of mechanical hypersensitivity was monitored. On day 26 post paclitaxel initiation, von Frey testing was performed on all rats and rats were then divided into two groups displaying similar levels of mechanical hypersensitivity. Rats then received an i.p. injection of 100 mg/kg PBN (n = 9) or an equivalent volume of vehicle (sterile 0.9% saline, n = 9). Rats were tested again for mechanical hypersensitivity at one hour, three hours and 24 hours following PBN/vehicle administration. This process was repeated for the next two consecutive days i.e. Day 27 & Day 28 post paclitaxel treatment. Thus rats with established paclitaxel-induced mechanical hypersensitivity received either three daily injections of 100 mg/kg PBN or saline, with each injection followed 1, 3 and 24 hours later by von Frey testing. Throughout the experiment, behavioural testing was performed under blind conditions by a single experimenter (MF). Injections were performed by another scientist. PBN/vehicle treatments were randomised within the equal groups of 6 animals being tested in a given session. These methods provided a concurrent vehicle-treated group throughout the experiments to control for potential variety in behavioural response due to the time of day. Following completion of the experiment, the identity of the treatment received by each rat was revealed and the data analysed.

#### Prophylactic dosing paradigm

Following habituation and four baseline measurements, rats were divided into two groups based on their responses to von Frey stimulation providing two groups with similar average baseline mechanical sensitivity. Rats received daily i.p. doses of either 50 mg/kg PBN (n = 8) or an equivalent volume of vehicle (sterile 0.9% saline, n = 8) for 15 consecutive days (day -1 through to day 13). On days 0, 2, 4 & 6 when paclitaxel was also administered, rats received PBN/vehicle injection before the paclitaxel injection. 50 mg/kg PBN was used in this experiment as opposed to 100 mg/kg due to concerns over tolerability to large injection volumes during an extended period. Mechanical sensitivity was then assessed in the mornings on days 7, 10, 14, 17, 19, 21, 25, 28, 31, 34, 38, 41 and 45 following the initiation of paclitaxel treatment (day 0). Throughout the experiment, behavioural testing and PBN/vehicle administration was performed under blind conditions by a single experimenter (MF). Following completion of the experiment, the identity of the treatment received by each rat was revealed and the data analysed.

### 2.4 TEMPOL experiments on mechanical hypersensitivity

4-Hydroxy-2,2,6,6-tetramethylpiperidine 1-oxyl (TEMPOL, Sigma-Aldrich, UK) dissolved in sterile 0.9% saline for injection (Fresenius Kabi, UK) resulting in an orange solution. TEMPOL was administered intraperitoneally (i.p.) in either a prophylactic or treatment dosing paradigm to test if TEMPOL could treat established paclitaxel-induced pain or prevent the development of paclitaxel-induced pain.

#### Treatment dosing paradigm

Following habituation and baseline testing, all rats received paclitaxel as described above and the emergence of mechanical hypersensitivity was monitored. On day 27 post paclitaxel initiation, von Frey testing was performed on all rats and rats were then divided into three groups displaying similar levels of mechanical hypersensitivity. Rats then received an i.p. injection of either 100 mg/kg TEMPOL (n = 8), 250 mg/kg TEMPOL (n = 8) or an equivalent volume of vehicle (sterile 0.9% saline, n = 8). Rats were tested again for mechanical hypersensitivity at one hour, three hours and 24 hours following TEMPOL/vehicle administration. Throughout the experiment, behavioural testing was performed under blind conditions by a single experimenter (SJLF). Injections were performed by another scientist. TEMPOL/vehicle treatments were randomised within the equal groups of 8 animals being tested in a given session. These methods provided a concurrent vehicle-treated group throughout the experiments to control for potential variety in behavioural response due to the time of day. Following completion of the experiment, the identity of the treatment received by each rat was revealed and the data analysed. Initially this TEMPOL treatment dosing paradigm was intended to run over three consecutive days (as performed for PBN). However due to significant side-effects (ptosis, pilorection, fits, and catatonia) observed immediately following 250 mg/kg administration the experiment was curtailed. These side-effects were not evident at the one hour testing time point.

#### Prophylactic dosing paradigm

In the prophylactic paradigm, following habituation and baseline testing, rats were divided into two groups based on their responses to von Frey stimulation providing two groups with similar baseline mechanical sensitivity. Rats received daily i.p. doses of either 100 mg/kg TEMPOL (n = 9) or an equivalent volume of vehicle (sterile 0.9% saline, n = 9) for 14 consecutive days (day −1 through to day 12). On days 0, 2, 4 & 6 when paclitaxel was also administered, rats received TEMPOL/vehicle injection before the paclitaxel injection. Mechanical sensitivity was then assessed in the mornings on days 7, 13, 17, 20, 24, 27, 33, 39 and 45 following the initiation of paclitaxel treatment (day 0). Throughout the experiment, behavioural testing and TEMPOL/vehicle administration was performed under blind conditions by a single experimenter (SJLF). Following completion of the experiment, the identity of the treatment received by each rat was revealed and the data analysed. As TEMPOL dissolved to give an orange solution, blinding procedures were more challenging than in PBN experiments. KCL Biological Services Unit staff kept SJLF blind to treatment received by randomisation of rat order within the elevated testing environment (at day 7) and by renumbering rats from day 12 onwards.

### 2.5 Behavioural assessment of cold hypersensitivity

Animals were placed on an elevated platform of small metal rods (spaced 8 mm apart) in individual Perspex boxes (dimensions 15 cm×16 cm×21 cm). Animals were allowed to acclimatise for 5–10 minutes before testing. Cold hypersensitivity was assessed using acetone, as previously described [31]. 50 µl of acetone was applied to the plantar surface of the hind paw using a Gilson P200 pipette and a stopwatch was started. In the following 20 seconds after acetone application the rat's response was monitored. If the rat did not withdraw, flick or stamp its paw within this 20-sec period then no response was recorded for that trial (0 points see below). However, if within this 20-sec period the animal responded to the cooling effect of the acetone, then the animal's response was assessed for an additional 20 seconds, a total of 40 seconds from initial application. Responses to acetone were graded to the following 4-point scale: **0** = no response, **1** = quick withdrawal, flick or stamp of the paw, **2** = prolonged withdrawal or repeated flicking (≥3) of the paw, **3** = repeated flicking of the paw with licking directed at the ventral side of the paw. Acetone was applied alternately three times to each paw and the responses scored categorically. At least 12 minutes had elapsed before the next application of acetone was applied to the same hind paw. Cumulative scores were then generated by adding the 6 scores for each rat together, the minimum score being 0 (no response to any of the 6 trials) and the maximum possible score being 18 (repeated flicking and licking of paws on each of the 6 trials). All testing was performed on rats when they were alert, not grooming and with all four paws in contact with the platform.

### 2.6 TEMPOL/PBN experiment on cold hypersensitivity

Following habituation and baseline testing, 24 rats received paclitaxel as described above and the emergence of cold hypersensitivity was monitored. Due to time constraints, this experiment using 24 rats was conducted on two separate days. On day 27 post paclitaxel initiation, acetone testing was performed on 15 rats and rats were then divided into three groups (n = 5) displaying similar levels of cold hypersensitivity. Rats then received an i.p. injection of either 100 mg/kg PBN, 100 mg/kg TEMPOL or an equivalent volume of vehicle (sterile 0.9% saline). Rats were tested again for cold hypersensitivity at one hour, three hours and 24 hours following PBN/TEMPOL/vehicle administration. On day 30 post paclitaxel initiation, acetone testing was performed on the remaining 9 rats and rats were then divided into three groups (n = 3) displaying similar levels of cold hypersensitivity. Similarly, rats then received an i.p. injection of either 100 mg/kg PBN, 100 mg/kg TEMPOL or an equivalent volume of vehicle (sterile 0.9% saline). Rats were tested again for cold hypersensitivity at one hour, three hours and 24 hours following PBN/TEMPOL/vehicle administration. Throughout the experiment, behavioural testing was performed under blind conditions by a single experimenter (LAG). Injections were performed by another scientist. PBN/TEMPOL/vehicle treatments were randomised within the groups of animals (7–9) being tested in a given session. These methods provided a concurrent vehicle-treated group throughout the experiments to control for potential variety in behavioural response due to the time of day for example. Following completion of the experiment, the identity of the treatment received by each rat was revealed and the data pooled from each part of the experiment resulting in n = 8 for each treatment group.

### 2.7 Statistics

**Figure 1:**
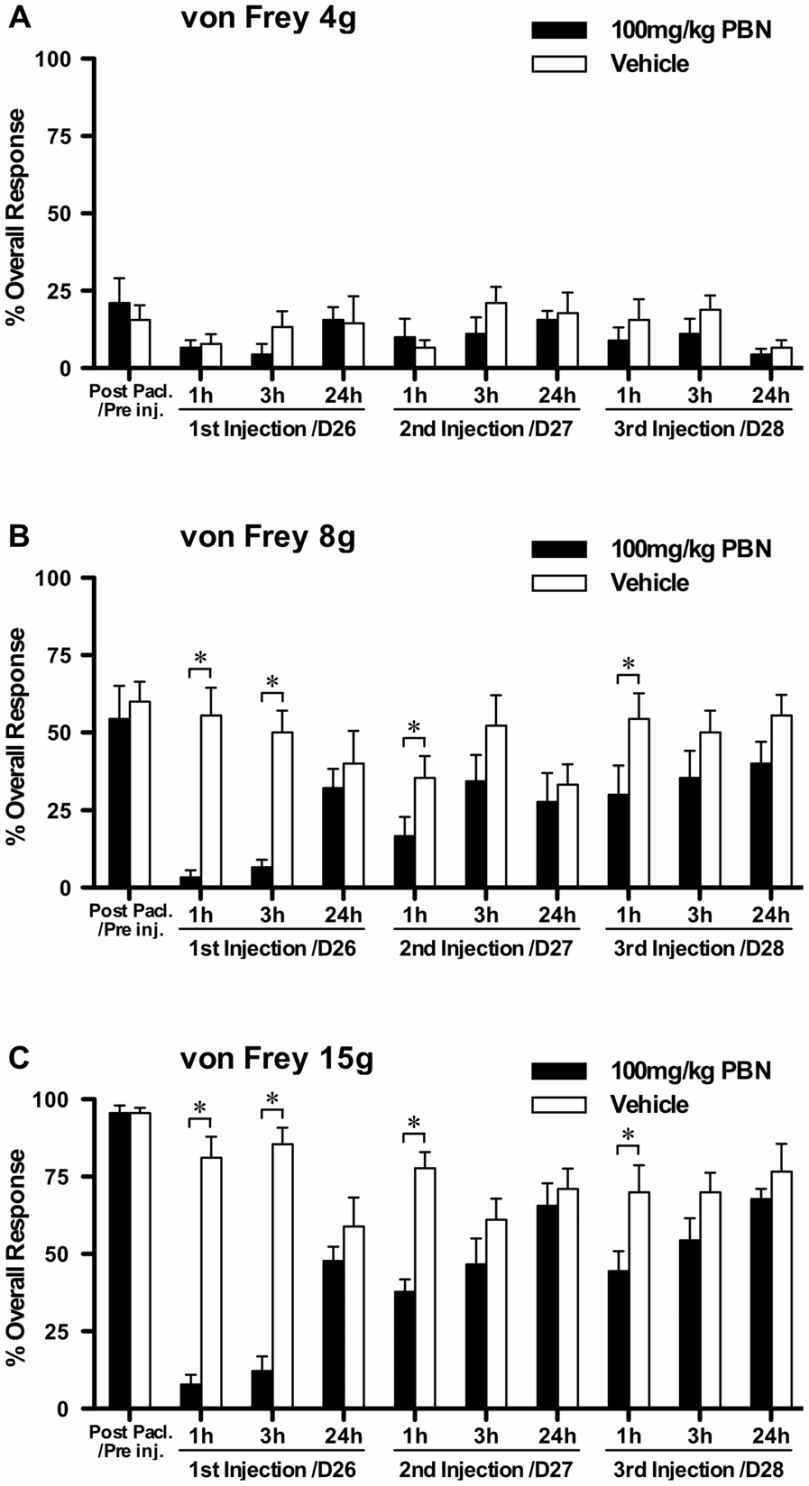
Effect of repeated PBN dosing on established paclitaxel-induced mechanical hypersensitivity. A–C show the effect of repeated systemic 100 mg/kg PBN or vehicle administration on behavioural responses to von Frey 4 g, 8 g and 15 g stimulation, respectively, on days 26, 27 & 28 following paclitaxel initiation. Graphs show the mean ± SEM of response frequency to mechanical stimulation after paclitaxel but before PBN/vehicle injection (Post Pacl./Pre inj.), and then following single doses of PBN/vehicle on consecutive days (1 h, 3 h and 24 h after PBN/vehicle injection). *p<0:05; one-tailed, unpaired t-tests with Bonferroni correction comparing PBN treatment to vehicle treatment at each time points, n = 9 per group.

**Figure 2:**
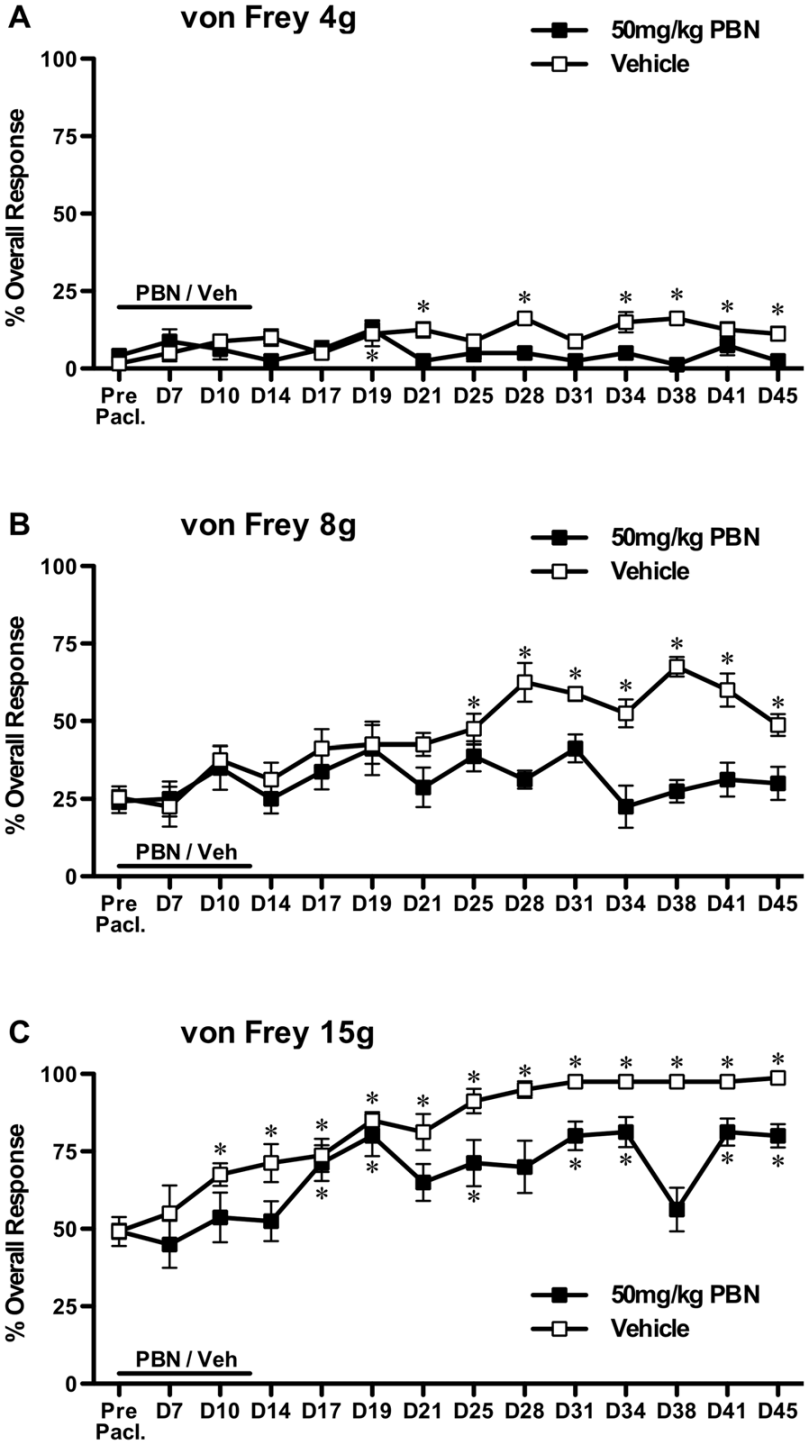
Effect of prophylactic PBN on the development of paclitaxel-induced mechanical hypersensitivity. Systemic 50 mg/kg PBN or vehicle was administered once daily for 15 consecutive days (day −1 through to day 13) with systemic paclitaxel administration occurring on days 0, 2, 4 & 6. Graphs show the mean ± SEM of the response frequency to mechanical stimulation by A) von Frey 4 g, B) von Frey 8 g and C) von Frey 15 g before paclitaxel (Pre Pacl.) and up to day 45 post-paclitaxel initiation. *p<0.05; one-way, repeated measures ANOVA with Dunnett's post hoc analysis compared to pre-paclitaxel readings, n = 8 per group. NB: The asterisks indicate the occurrence of significant paclitaxel-induced mechanical hypersensitivity.

**Figure 3:**
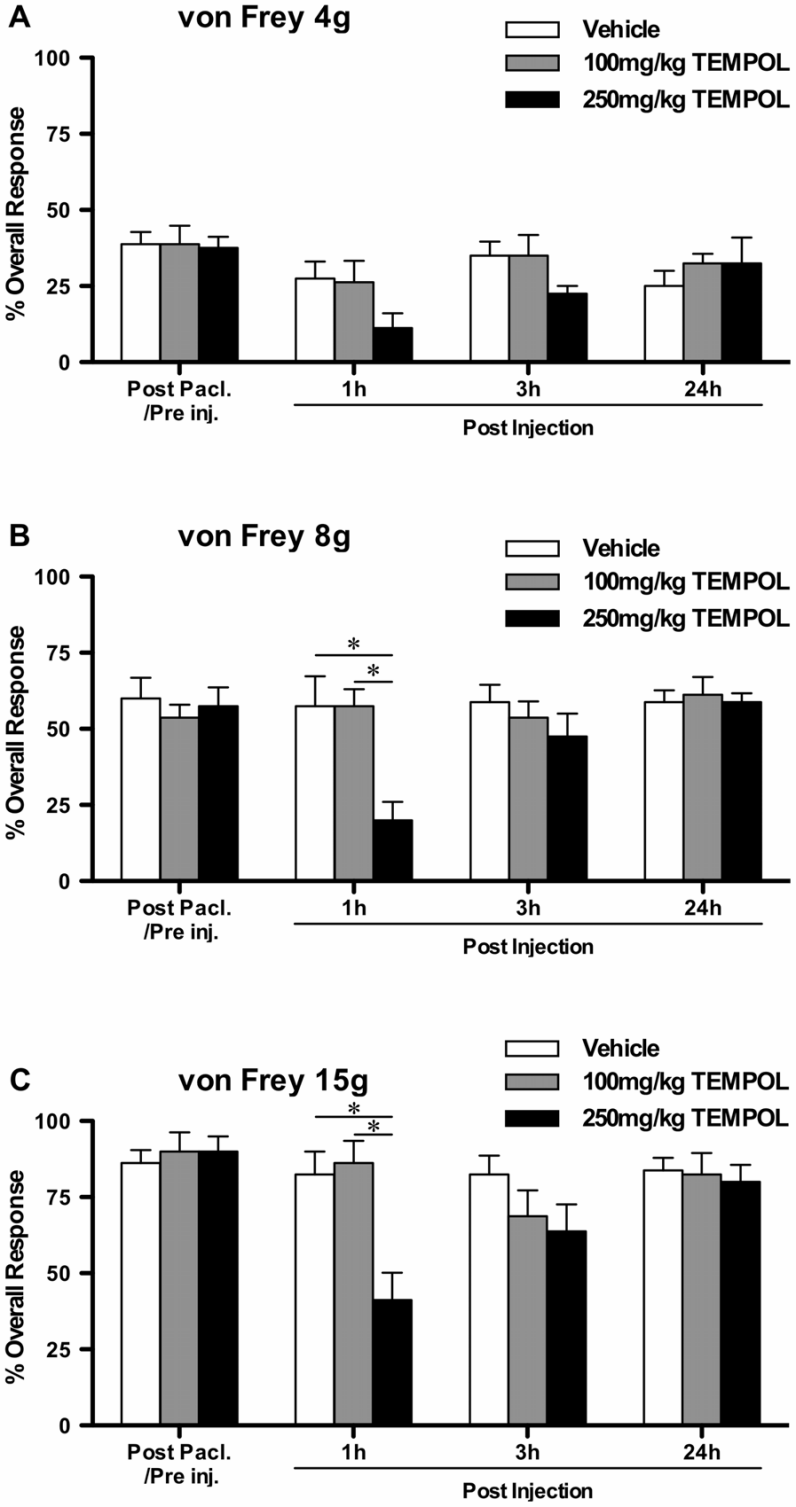
Effect of TEMPOL on established paclitaxel-induced mechanical hypersensitivity. A–C show the effect of systemic 100 mg/kg TEMPOL, 250 mg/kg TEMPOL or vehicle administration on behavioural responses to von Frey 4 g, 8 g and 15 g stimulation, respectively, on day 27 following paclitaxel initiation. Graphs show the mean ± SEM of response frequency to mechanical stimulation after paclitaxel but before TEMPOL/vehicle injection (Post Pacl./Pre inj.), and then 1, 3 and 24 hours following TEMPOL/vehicle administration. *p<0:05; one-way ANOVA with Tukey-Kramer post hoc analysis, n = 8 per group.

**Figure 4:**
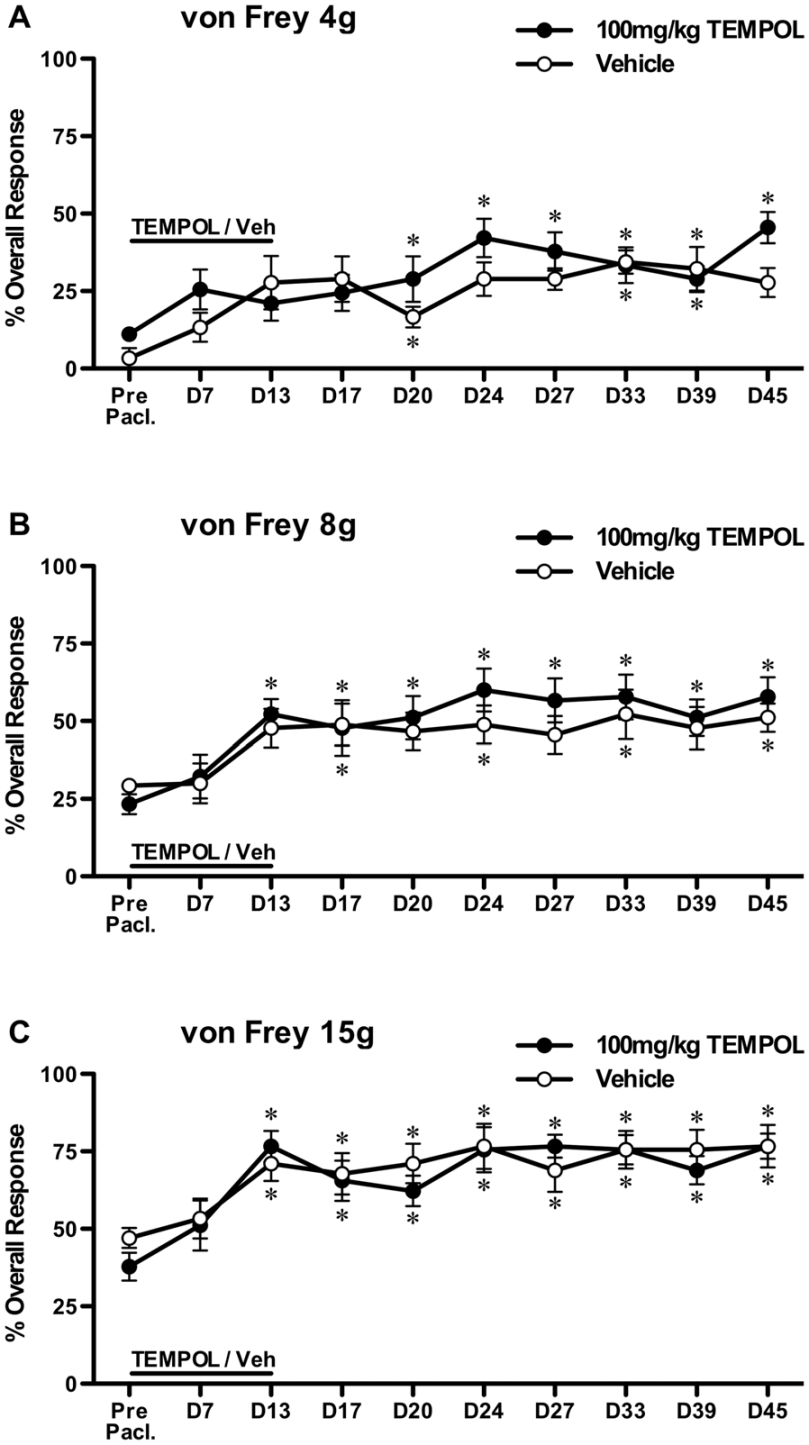
Effect of prophylactic TEMPOL on the development of paclitaxel-induced mechanical hypersensitivity. Systemic 100 mg/kg TEMPOL or vehicle was administered once daily for 14 consecutive days (day −1 through to day 12) with systemic paclitaxel administration occurring on days 0, 2, 4 & 6. Graphs show the mean ± SEM of the response frequency to mechanical stimulation by A) von Frey 4 g, B) von Frey 8 g and C) von Frey 15 g before paclitaxel (Pre Pacl.) and up to day 45 post-paclitaxel initiation. *p<0.05; one-way, repeated measures ANOVA with Dunnett's post hoc analysis compared to pre-paclitaxel readings, n = 9 per group. NB: The asterisks indicate the occurrence of significant paclitaxel-induced mechanical hypersensitivity.

**Figure 5:**
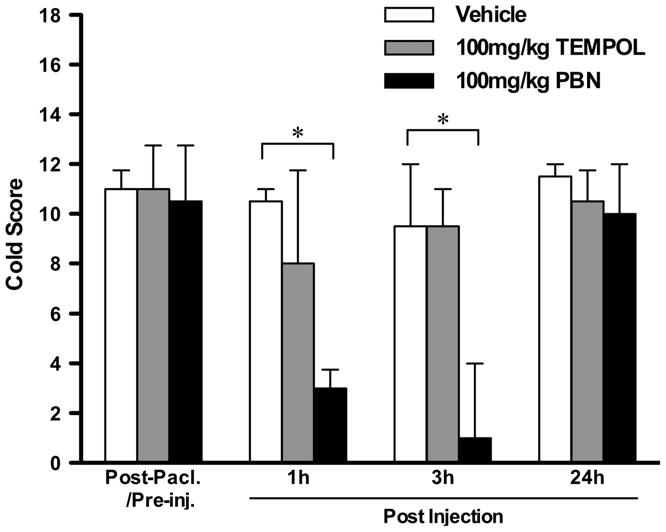
Effect of PBN or TEMPOL on established paclitaxel-induced cold hypersensitivity. Graph shows the effect of systemic 100 mg/kg PBN, 100 mg/kg TEMPOL or vehicle administration on behavioural responses to acetone stimulation on day 27/30 following paclitaxel initiation. Data shown is the median cold score ± interquartile range after paclitaxel but before PBN/TEMPOL/vehicle injection (Post Pacl./Pre inj.), and then 1, 3 and 24 hours following administration of PBN/TEMPOL/vehicle. *p<0.05; Kruskal-Wallis with Dunn's Multiple Comparisons post-hoc analysis compared to vehicle-treated control, n = 8 per group.

One tailed unpaired t-tests with Bonferroni correction were used to compare the effects of repeated PBN treatment to vehicle treatment on established paclitaxel-induced mechanical hypersensitivity (Fig. 1). One way, repeated measures, analysis of variance (ANOVA) followed by Dunnett's post hoc analysis was used to test for significant development of paclitaxel-induced mechanical hypersensitivity following prophylactic administration of PBN/vehicle (Fig. 2). One tailed unpaired t-tests with Bonferroni correction were also used to compare prophylactic PBN-treated group responses to von Frey 15 g to vehicle-treated group responses (Fig. 2C). One way ANOVA with Tukey-Kramer post hoc analysis was used to compare effects of 100 mg/kg & 250 mg/kg TEMPOL to concurrent vehicle-treated group on established paclitaxel-induced mechanical hypersensitivity (Fig. 3). One way, repeated measures, analysis of variance (ANOVA) followed by Dunnett's post hoc analysis was used to test for significant development of paclitaxel-induced mechanical hypersensitivity following prophylactic administration of TEMPOL/vehicle (Fig. 4). Kruskal-Wallis test with Dunn's multiple comparisons post hoc analysis was used to compare effects of PBN and TEMPOL to the concurrent vehicle-treated group on established paclitaxel-induced cold hypersensitivity (Fig. 5). Statistical significance was accepted at p<0.05. No distinction has been made when p<0.01 or p<0.001 and is denoted on figures as p<0.05.

## Results

A cumulative dose of 8 mg/kg paclitaxel administered in four i.p. injections resulted in significant mechanical and cold hypersensitivity, assessed by responses to von Frey 4 g, 8 g and 15 g stimulation and acetone application, respectively. Maximal mechanical hypersensitivity was observed around day 27 post paclitaxel initiation as previously described [28]. Figure 1 shows the effect of repeated systemic 100 mg/kg PBN administration on maximal paclitaxel-induced mechanical hypersensitivity. Responses to von Frey 4 g were unaffected by PBN treatment throughout the experiment (Fig. 1A). In contrast, PBN significantly inhibited responses to von Frey 8 g & 15 g, by 52%–73%, at one and three hours following the first injection compared to the concurrent vehicle treated group at those time points (p<0.05, Figs. 1B&C, one-tailed unpaired t-tests with Bonferroni correction). Subsequent systemic PBN administration on the following two days also significantly inhibited responses to von Frey 8 g & 15 g one hour post injection only by 19% and 40%, respectively, compared to the concurrent vehicle treated group at that time point (p<0.05, Figs. 1B&C, one-tailed unpaired t-tests with Bonferroni correction). This smaller inhibitory effect on repeated dosing could indicate a degree of tolerance to systemic PBN treatment. Pre-paclitaxel responses to von Frey stimulation, in the rats used in this experiment, were very similar to those responses shown in Figure 2 (Pre Pacl.).

Prophylactic PBN administration before, during and after paclitaxel administration (50 mg/kg daily, day −1 through to day 13) had a marked preventative effect on the development of paclitaxel-induced mechanical hypersensitivity (Figure 2). No significant increase was observed in the responses to von Frey 4 g & 8 g of rats that received PBN treatment at any time point compared to pre-paclitaxel readings (Fig. 2 A&B, one-way repeated measures ANOVA with Dunnett's post hoc analysis). In comparison, a progressive increase in von Frey 4 g & 8 g responses was seen in vehicle-treated rats that were statistically significant from day 19 and 25, respectively, compared to pre-paclitaxel readings (p<0.05, Fig. 2 A&B, one-way repeated measures ANOVA with Dunnett's post hoc analysis). Responses to von Frey 15 g were significantly increased compared to pre-paclitaxel levels in rats that had received either PBN or vehicle over the time course (p<0.05, Fig. 2C, one-way repeated measures ANOVA with Dunnett's post hoc analysis). However, the magnitude of paclitaxel-evoked increase in overall response to von Frey 15 g in relation to pre-paclitaxel responses was 32% in the PBN group compared to 48% in the vehicle group. Furthermore, significant hypersensitivity to von Frey 15 g in the vehicle group was first observed at day 10 and present for 12 of 13 time points, whereas in the PBN group it was first observed at day 17 and present for 7 of 13 time points (Fig. 2C). Direct statistical comparison of von Frey 15 g responses between prophylactic vehicle and PBN administration showed that PBN caused a significant attenuation at day 31, 38 and 45 (p<0.05, one-tailed unpaired t-tests with Bonferroni correction).

Given these inhibitory effects on paclitaxel-induced pain of global ROS inhibition by PBN, we investigated whether these inhibitory effects could be replicated with selective inhibition of superoxide radicals (O_2_^−^) using TEMPOL, a superoxide dismutase mimetic. Figure 3 shows the effects of systemic 100 mg/kg and 250 mg/kg TEMPOL on maximal paclitaxel-induced mechanical hypersensitivity. 100 mg/kg TEMPOL had no effect on responses to 4 g, 8 g or 15 g von Frey stimulation either at one hour, three hours or 24 hours post injection (Fig. 3, one-way ANOVA with Tukey-Kramer post hoc analysis). Similarly, von Frey 4 g responses were unaffected by 250 mg/kg TEMPOL. However, 250 mg/kg TEMPOL significantly inhibited responses to von Frey 8 g & 15 g at one hour post injection by 38% and 41%, respectively, compared to the concurrent vehicle-treated group (p<0.05, Fig. 3, one-way ANOVA with Tukey-Kramer post hoc analysis). Side-effects were observed 1–5 minutes following 250 mg/kg TEMPOL administration; all rats showed ptosis, 6 of 8 rats were markedly subdued and 3 of 8 rats showed jerking movements and shaking. There was no evidence of these effects during von Frey testing at one, three or 24 hours post administration. However additional days of dosing such as those in the PBN experiment were not performed due to concerns regarding tolerability to repeated 250 mg/kg TEMPOL dosing.

The effects of prophylactic TEMPOL administration before, during and after paclitaxel administration (100 mg/kg daily, day −1 through to day 12) are illustrated in Figure 4. A significant mechanical hypersensitivity to von Frey 4 g, 8 g & 15 g stimulation developed in both TEMPOL and vehicle-treated groups in a similar manner. A significantly increased response to von Frey 4 g stimulation was observed from day 20 onwards, in TEMPOL and vehicle-treated groups, comparing to pre-paclitaxel response levels (p<0.05, Fig. 4A, one-way repeated measures ANOVA with Dunnett's post hoc analysis). Significantly increased responses to von Frey 8 g & 15 g stimulation were observed from day 13 onwards, in TEMPOL and vehicle-treated groups, comparing to pre-paclitaxel response levels (p<0.05, Fig. 4B&C, one-way repeated measures ANOVA with Dunnett's post hoc analysis). There was no significant difference in responses to von Frey stimulation following prophylactic TEMPOL or vehicle administration, at any time point.

Paclitaxel is also known to induce cold hypersensitivity in humans [5] and rats [31]. Therefore we assessed the effects of PBN and TEMPOL on established paclitaxel-induced cold hypersensitivity to compare to their effects on mechanical hypersensitivity. Figure 5 shows the effects of systemic 100 mg/kg PBN and 100 mg/kg TEMPOL on maximal paclitaxel-induced cold hypersensitivity. PBN significantly inhibited responses to acetone application at one and three hours post administration compared to vehicle administration (p<0.05, Figure 5, Kruskal-Wallis test with Dunn's multiple comparisons post hoc analysis). In contrast, TEMPOL had no effect on paclitaxel-induced cold hypersensitivity.

## Discussion

In this study, we have examined the role of ROS in the maintenance and development of paclitaxel-induced painful peripheral neuropathy *in vivo*. We have observed the effects of systemic administration of a non-specific ROS scavenger, PBN, and a superoxide-specific scavenger, TEMPOL, in both treatment and prophylactic dosing paradigms. The rationale for these experiments was to test if PBN and/or TEMPOL could reverse established paclitaxel-induced pain and/or prevent the development of paclitaxel-induced pain, as both scenarios have significant clinical relevance.

The first administration of PBN markedly inhibited established paclitaxel-induced mechanical hypersensitivity to von Frey 8 g and 15 g stimulation for over three hours. Similarly, repeated bolus PBN treatment on the following two days also significantly inhibited this mechanical hypersensitivity, but to a lesser extent, perhaps indicating tolerance to repeated PBN administration. Mechanical hypersensitivity to von Frey 4 g was unaffected by PBN treatment, which could suggest a lack of anti-allodynic effect by PBN or the relatively small window of hypersensitivity to von Frey 4 g in this cohort of rats to elicit a statistically significant inhibition. PBN also significantly inhibited paclitaxel-induced cold hypersensitivity. In comparison, prophylactic PBN dosing completely prevented the development of mechanical hypersensitivity to von Frey 4 g & 8 g stimulation through to day 45 post paclitaxel initiation. Prophylactic PBN dosing also delayed the appearance of, and reduced the magnitude of mechanical hypersensitivity to von Frey 15 g. These effects demonstrate that ROS play a role in both the maintenance and development of paclitaxel-induced pain.

The effects of TEMPOL on paclitaxel-induced mechanical hypersensitivity we observed were quite different to the inhibitory effects of PBN. The same dose of TEMPOL (100 mg/kg) did not inhibit established paclitaxel-induced mechanical hypersensitivity. High dose (250 mg/kg) TEMPOL inhibited mechanical hypersensitivity to von Frey 8 g and 15 g at one hour, but to a lesser extent than observed with PBN and these effects were not present at three hours post administration. Similar to PBN, TEMPOL had no effect on established mechanical hypersensitivity to von Frey 4 g. However, in marked contrast to PBN, prophylactic TEMPOL showed no inhibitory effects on the development of paclitaxel-induced mechanical hypersensitivity through to day 45 post paclitaxel initiation. Prophylactic TEMPOL was administered at twice the prophylactic PBN dose that prevented development of paclitaxel-induced mechanical hypersensitivity. It is possible that higher doses of TEMPOL may have an inhibitory effect on the development of paclitaxel-induced pain. However, given the side-effects observed in the minutes following 250 mg/kg TEMPOL administration in the established pain study, we decided that 100 mg/kg was the maximally tolerated dose for repeated prophylactic dosing. The overall lack of effect of TEMPOL in this study suggests that superoxide radicals do not play a role in the maintenance or development of paclitaxel-induced pain. The lack of parallel effects of PBN and TEMPOL in paclitaxel-induced pain provides further evidence that chemotherapy-induced painful peripheral neuropathies have different causal mechanisms to other pain states. Previously, both PBN and TEMPOL at similar doses have been shown to inhibit SNL-evoked heat and mechanical hypersensitivity in a similar manner [20]. Furthermore, both PBN and TEMPOL inhibited capsaicin-induced secondary hyperalgesia when administered systemically before or after capsaicin administration [22].

A recent study has examined the effects of PBN on paclitaxel-induced mechanical hypersensitivity [33]. These authors used the same dosing schedule of paclitaxel (2 mg/kg i.p. on days 0, 2, 4 & 6) as used in this study, although the paclitaxel was dissolved in a vehicle solution of DMSO, Tween 80 and saline as opposed to the clinical formulation (Cremophor EL, ethanol and saline) used here. On established paclitaxel-induced mechanical hypersensitivity they found, as we report here, inhibitory effects following single and repeated systemic administration of 100 mg/kg PBN. They also examined prophylactic PBN dosing paradigms on the development of paclitaxel-induced mechanical hypersensitivity. Daily 200 mg/kg PBN administration on days 0–7 had no effect on the development of paclitaxel-induced mechanical hypersensitivity, whereas daily 200 mg/kg PBN administration on days 7–15 prevented the development of paclitaxel-induced mechanical hypersensitivity [33]. In comparison, here we have shown that daily 50 mg/kg PBN administration for two weeks starting one day before the first dose of paclitaxel (day −1) through to day 13 prevented the development of mechanical hypersensitivity to von Frey 4 g and 8 g and significantly attenuated mechanical hypersensitivity to von Frey 15 g. Comparing our results to Kim et al., suggests that lower doses of PBN over a longer dosing period that start prior to paclitaxel exposure could be as effective as much higher doses of PBN after the paclitaxel exposure. Alternatively, the timing of systemic PBN administration in relation to the paclitaxel administration could be more functionally important than dosage. In this case, comparison of the two studies suggests that the week immediately following the end of the paclitaxel dosing (day 7–13) is the crucial period to prevent the emergence of paclitaxel-induced mechanical hypersensitivity. Therefore, potentially paclitaxel-induced ROS during this time period evokes changes in the nociceptive system that initiate the paclitaxel-induced pain syndrome, shown to persist for 5 months in this model [28].

The causal mechanism(s) for chemotherapy-induced painful peripheral neuropathy are unclear. Various rodent models of paclitaxel-induced painful peripheral neuropathy have been reported using different systemic dosing schedules and cumulative doses of paclitaxel [30], [34], [35], [36], [37], [38]. The consensus of these studies is similar to the clinical scenario, in that the degree of degeneration observed increases according to the amount of paclitaxel administered. The low doses of paclitaxel used here to evoke paclitaxel-induced pain, do not cause axonal degeneration demonstrated by morphological analysis of peripheral nerves and ATF3 expression in dorsal root ganglia [28], [30]. However atypical (swollen and vacuolated) mitochondria in peripheral sensory axons [28], [39] and a loss of intraepidermal nerve fibres [40], [41] occur as the paclitaxel-induced pain syndrome develops. These pathological changes can be prevented pharmacologically in concert with the paclitaxel-induced pain syndrome suggesting a causal role for both phenomena in the aetiology of paclitaxel-induced painful peripheral neuropathy. Acetyl-L-carnitine has been shown to prevent the development of paclitaxel-induced pain [32] and the paclitaxel-induced increase in atypical mitochondria in C-fibres, but not the paclitaxel-induced loss of intraepidermal nerve fibres [39]. Minocycline has been shown to prevent the development of paclitaxel-induced pain [42] and prevent paclitaxel-induced loss of intraepidermal nerve fibres [41].

The inhibitory effects of a non-specific ROS scavenger reported here indicate that ROS has a causal role in paclitaxel-induced pain. While we have provided evidence to suggest superoxide radicals do not play a major role in paclitaxel-induced painful peripheral neuropathy, further study could address the contribution of other free radicals such as hydrogen peroxide, peroxynitrite and hydroxyl radicals. Considering that mitochondria are a major source of ROS, this perhaps demonstrates a consequential mechanism of how the atypical mitochondria in peripheral sensory axons lead to paclitaxel-induced pain. Alternatively, as superoxide radicals are predominantly derived from mitochondria, it is possible that the ROS responsible for paclitaxel-induced pain are generated from other sites in the cell. In this model of paclitaxel-induced painful peripheral neuropathy, atypical mitochondria were observed at day 7 (where no pain behaviour is observed) and day 27 (peak pain severity) [28] and PBN administration between days 7–13 appears to prevent the emergence of paclitaxel-induced mechanical hypersensitivity as previously discussed. This could open up the intriguing possibility that the paclitaxel-induced increase in atypical mitochondria is a consequence of ROS generation as opposed to the cause of ROS generation.

In conclusion, this study demonstrates that global inhibition of ROS can inhibit established paclitaxel-induced pain and prevent the development of paclitaxel-induced pain, whereas selective inhibition of superoxide radicals was mostly ineffective. The causal role of ROS in paclitaxel-induced painful peripheral neuropathy highlights a potential novel therapeutic strategy for the prevention and treatment of this major dose-limiting side-effect.
